# Statins Attenuate the Increase in P-Selectin Produced by Prolonged Exercise

**DOI:** 10.1155/2013/487567

**Published:** 2013-05-07

**Authors:** Amanda Zaleski, Jeffrey Capizzi, Kevin D. Ballard, Christopher Troyanos, Aaron Baggish, Pierre D'Hemecourt, Paul D. Thompson, Beth Parker

**Affiliations:** ^1^Henry Low Heart Center, Department of Cardiology, Hartford Hospital, Hartford, CT 06102, USA; ^2^Department of Kinesiology, University of Connecticut, Storrs, CT 06269, USA; ^3^Children's Hospital, Boston, MA 02115, USA; ^4^Division of Cardiology, Massachusetts General Hospital, Boston, MA 02114, USA

## Abstract

Strenuous endurance exercise increases inflammatory markers and acutely increases cardiovascular risk; however, statins may mitigate this response. We measured serum levels of p-selectin in 37 runners treated with statins and in 43 nonstatin treated controls running the 2011 Boston Marathon. Venous blood samples were obtained the day before (PRE) as well as within 1 hour after (FINISH) and 24 hours after (POST) the race. The increase in p-selectin immediately after exercise was lower in statin users (PRE to FINISH: 20.5 ± 19.4 ng/mL) than controls (PRE to FINISH: 30.9 ± 27.1 ng/mL; *P* < 0.001). The increase in p-selectin 24 hours after exercise was also lower in statin users (PRE to POST: 21.5 ± 26.6 ng/mL) than controls (PRE to POST: 29.3 ± 31.9 ng/mL; *P* < 0.001). Furthermore, LDL-C was positively correlated with p-selectin at FINISH and POST (*P* < 0.01 and *P* < 0.05, resp.), irrespective of drug treatment, suggesting that lower levels of LDL-C are associated with a reduced inflammatory response to exercise. We conclude that statins blunt the exercise-induced increase in p-selectin following a marathon and that the inflammatory response to a marathon varies directly with LDL-C levels.

## 1. Introduction 

We recently showed that p-selectin, a biomarker greatly associated with coronary disease and inflammation, increased after a marathon [1]. Serious cardiac events have also rarely been associated with marathon running [2], and increases in such biomarkers of thrombotic and vascular risk such as p-selectin may contribute to this increased cardiac risk [3].

 HMG CoA reductase inhibitors (statins) are the most effective medicines for lowering LDL cholesterol and substantially reduce cardiovascular disease mortality. However, statins evoke many other pleiotropic effects, including anti-inflammatory actions, which could also contribute to their reducing cardiovascular events [4]. The present study determined whether chronic statin therapy ameliorates the inflammatory response to endurance exercise. We hypothesized that statin treated runners would exhibit lower p-selectin levels immediately and 24 hours after the marathon than nonstatin using runners. 

## 2. Methods

 Thirty seven statin-using athletes (29 men and 8 women) and 43 controls (30 men and 13 women) were recruited through an email sent to all participants registered for the 115th Boston Athletic Association Marathon held on April 18, 2011. Subjects were recruited if they either had continuously received statin therapy for >6 months or had not used any lipid-lowering medication. Subjects were nonsmokers, ≥age 35, and free of known cardiovascular or metabolic disease besides hypercholesterolemia. Subjects were not taking oral contraceptives and/or hormone therapy and agreed to abstain from taking any nonstatin medications for 24 hours before the race that could affect blood biomarkers. This included aspirin and nonsteroidal anti-inflammatories (NSAIDS). Subjects provided written, informed consent to participate as approved by the Institutional Review Board at Hartford Hospital.

The day before the marathon subjects presented to a room provided by the Boston Athletic Association at the marathon exposition. Subjects provided a medical history and reported their training mileage over the 3 months and week preceding the marathon. Resting blood pressure and heart rate (Welch Allen 52000 Vital Signs Monitor; Skaneateles Falls, NY) as well as height and body mass were measured. Venous blood was obtained after a 12-hour fast to measure total and high density lipoprotein (HDL-C) cholesterol and triglycerides. Low density lipoprotein cholesterol (LDL-C) was estimated using the Friedewald equation [5]. Plasma was separated from cells by centrifugation at 5000 r.p.m. for 10 minutes and stored on dry ice (−80°C). Samples were shipped to Quest Diagnostics Nichols Institute, Chantilly, VA, where blood lipid analyses were performed. Blood was also obtained immediately after the subjects completed the marathon in the main medical tent approximately 100 meters from the finish line and the day after the race (within 24 hours of the finish) at the subjects nearest Quest Diagnostics location. Cryovials obtained remotely were shipped overnight to Hartford Hospital on dry ice and archived in a −80 freezer. Plasma from each measurement point was analyzed for soluble p-selectin. 

 Soluble p-selectin was measured in duplicate using enzyme-linked immunosorbent assays (R&D Systems, Minneapolis, Minnesota) (coefficient of variation 3.6%). For all assays, absorbance was determined on a spectrophotometer (VersaMax Microplate Reader, Molecular Devices, Sunnyvale, California), and data were analyzed using associated software (SoftMax Pro Microplate Data Acquisition and Analysis software, version 5.3, Molecular Devices, Sunnyvale, CA). 

Differences in baseline characteristics between statin and control groups were assessed with a one-way analysis of variance (ANOVA) with significance set at *P* < 0.05. To determine the effects of statin use on changes in p-selectin, we used a linear mixed model for repeated measurements with autoregressive variance-covariance structure, incorporating time as the within-subjects factor and group (control versus statin) as the between-subjects factor. Subjects were defined as the random factor; all other variables were fixed within the model. Potential categorical factors that could affect the relation between the main effects and outcomes were added into the model to assess significance, and the effect of continuous variables was investigated using analysis of covariance (ANCOVA). *P* values for mean difference estimates between groups at various time points were adjusted using Tukey's multiple comparison procedure to account for post-hoc multiple comparison testing. Pearson correlations and linear regression were used to examine the relationships between continuous variables. To investigate the effect of statin potency in statistical models, statins were classified by expected potency of cholesterol reduction according to published dose equivalencies: rosuvastatin 2.5 mg = atorvastatin 5 mg = simvastatin 10 mg = lovastatin 20 mg = pravastatin 20 mg = fluvastatin 40 mg [6, 7]. Cross-tabulation and chi-squared analyses were performed to examine the frequency distribution of p-selectin among established risk quartiles [8].

Statistical analyses were performed with SAS 9.1 (Cary, NC), and all data are expressed as nontransformed values are presented in the tables and figures with group means ± standard deviation (SD).

## 3. Results

### 3.1. Subject Characteristics

The baseline characteristics between the statin and control group subjects have been previously published [9]. Statin users were slightly older, had lower LDL-C and HDL-C values (Table 1), and were treated with a variety of statins and statin doses (Table 2). Six different types of statins were normalized for potency using published dose equivalencies [6, 7]. The average potency of statin used by participants in atorvastatin equivalents was 14.7 mg. Potency was inversely related to total and LDL-C in the statin group (Pearson coefficients = −0.37 and −0.36, resp., and both *P* < 0.05), but not related to p-selectin PRE, FINISH, or POST (*P* > 0.05). Three statin participants reported using niacin 500–1000 mg.

**Figure 1:**
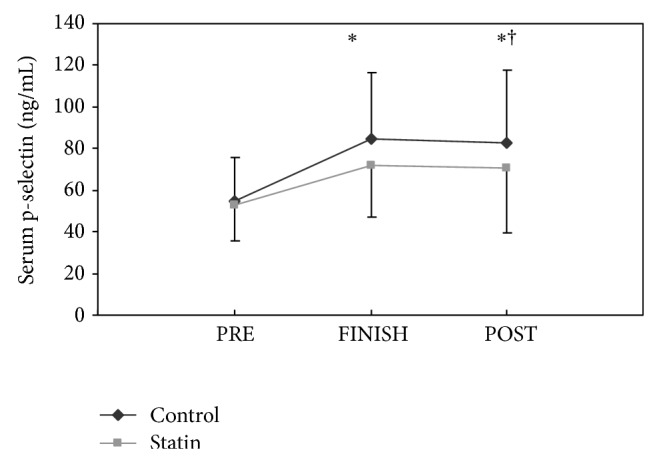
Group means (± SD) of serum p-selectin before (PRE), immediately after (FINISH), and 24 hours after the marathon (POST) in statin users versus controls. ∗Significant change relative to the baseline (PRE) value at *P* < 0.001 within each group and † denotes a significant difference between groups at *P* < 0.05.

P-selectin levels prior to the marathon were similar in the statin users and controls and similar to resting levels reported in studies with healthy normal controls [8] (Table 1). Exercise-induced increases in p-selectin were observed in both groups; however, the increase in p-selectin immediately after exercise was lower in statin users when controlled for significant covariates BMI, age, and HDL (Figure 1). The increase in p-selectin 24 hours after exercise was also lower in statin users (Figure 1). There were no significant main effects or interactions among exercise-induced increases in p-selectin, age, or gender.

**Figure 2:**
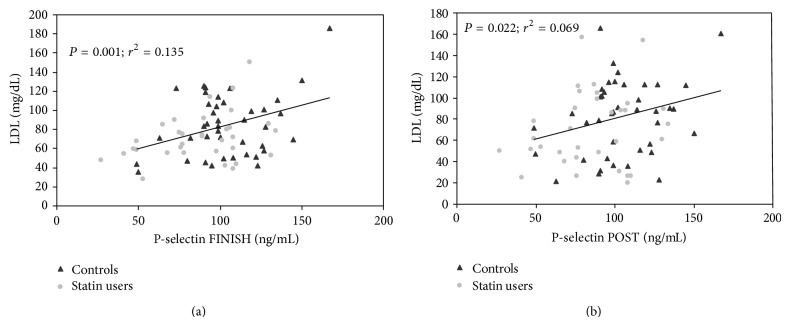
Relationship between LDL and p-selectin immediately after (FINISH) the marathon and 24 hours (POST) after the marathon in total group.

LDL was positively correlated with p-selectin at FINISH and POST (*P* < 0.01 and *P* < 0.05, resp.), but not at PRE (*P* = 0.16) (Figure 2). 

**Figure 3:**
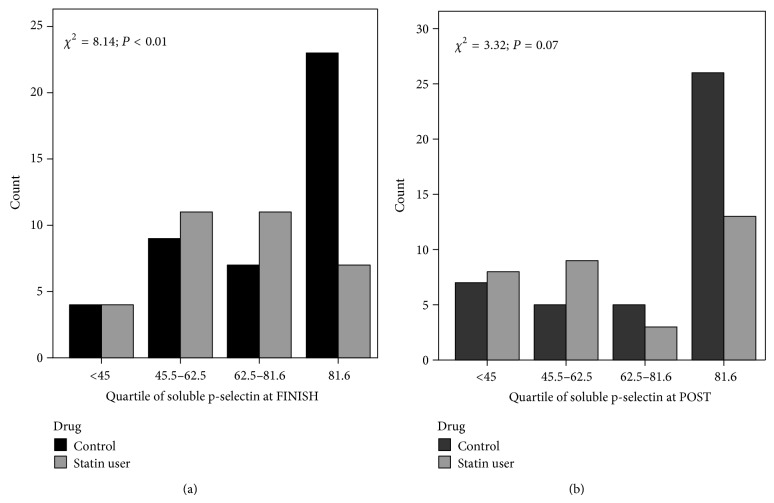
Number of cases in each quartile of soluble p-selectin after (FINISH) the marathon and 24 hours (POST) after the marathon in statin users versus controls.

We also examined the estimated risk of future cardiovascular events using established quartiles according to p-selectin levels measured at FINISH and POST [8]. Immediately following the marathon (FINISH) the distribution of runners with a relative risk quartile above the >4th quartile cut point was significantly higher in controls versus statin using athletes (23 control versus 7 statin users; *χ* ^2^ = 8.14; *P* < 0.01) (Figure 3). The day following the marathon (POST) controls tended to have a higher distribution of runners in the 4th quartile when compared to statin users; however, this trend did not reach statistical significance (26 control versus 13 statin users) (*P* = 0.07).

## 4. Discussion 

The present study investigated the effect of chronic statin therapy on exercise-associated increases in p-selectin. We have documented that a marathon run increases p-selectin in both controls and statin using athletes (Figure 1). These findings are consistent with other studies [10, 11] as well as our previously published results from the 2010 Boston Marathon, in which we observed a similar increase in p-selectin immediately and 24 hours following a marathon in nonstatin using runners [1]. 

Importantly, however, the increase in p-selectin was lower in statin users than controls when we adjusted for significant covariates of BMI, age, and HDL. To the best of our knowledge, this is the first such study investigating the effects of statin therapy on exercise-associated increases in p-selectin in healthy adults; however, findings are consistent with data indicating that statins reduce p-selectin in patients with coronary artery disease [12] and hypercholesterolemia [13] unrelated to vigorous exercise. 

We have previously noted that the increase in p-selectin immediately following a marathon could increase the risk of a cardiovascular event during the marathon [1]. Ridker et al. [8] have previously shown that the relative risk of future cardiovascular events increases by 27% for each quartile increase in p-selectin. For example, in our study of Boston Marathon participants not using statins, twelve runners at finish and 10 runners the day following the marathon had p-selectin values >45.5 ng/mL (the second quartile), increasing their estimated risk of a cardiovascular event by 27%. In the current study of older runners, the number of runners with p-selectin values exceeding 81.6 ng/mL (the highest quartile of risk) immediately following the marathon was significantly higher in controls than in statin using runners (23 control versus 7 statin) (Figure 3). 

 The lower levels of p-selectin seen among runners using statins may be due to statins reducing the upregulation of the inflammatory response produced by endurance running [14, 15]. P-selectin, a cell adhesion receptor, is stored within *α*-granules of platelets and Weibel-Palade bodies of endothelial cells [16]. Cytokine stimulation produces a translocation of p-selectin from the intracellular platelets and endothelial cells to the surface of endothelial cells, which mediates inflammation and platelet aggregation [17]. Inhibition of HMG-CoA reductase via statin therapy activates the mevalonate pathway, which initiates downstream isoprenylation and subsequent depletion of cytokines such as interleukin (IL) and TNF*α* [18–20].

 We have previously noted that age is positively correlated with p-selectin levels before and after the marathon [1], possibly contributing to the greater risk of an adverse CV event in older runners [2]. However, in the present study we did not observe a significant interaction between baseline or exercise-induced increases in p-selectin and age in either group. This could be due to the older age of the current sample, as other factors including lipid levels [21], vascular stiffness [21], BMI [22] may influence p-selectin levels in older adults. 

 Indeed, independent of statin therapy, LDL was positively correlated with p-selectin immediately after and the day following the marathon (Figure 2), such that runners with the lowest LDL levels also exhibited the lowest postexercise p-selectin values. Platelet-bound oxLDL is positively correlated with expression of p-selectin and subsequently platelet aggregation. Therefore, in runners who do not achieve low LDL via nonpharmacologic therapies, statin therapy may be a potential therapeutic intervention with which to mitigate the risk of marathon related cardiovascular events. 

There are limitations to the present study. Statin use was based solely on self-report, but calculated statin potency was inversely associated with LDL cholesterol levels, supporting the accuracy of subject self-reports. Subjects used a variety of statins and doses making it difficult to determine if a dose response effect occurs with low versus high dose statins. There was not a correlation between potency and p-selectin as we did not recruit based on statin type and dosage and consequently were not powered to detect differences. We were unable to recruit sufficient numbers of women to explore gender differences in the p-selectin response to exercise with statins. This would be interesting to explore as male marathon runners have increased incidence of cardiac arrest when compared to women [2].

There are several clinical implications to the present results. Marathon related cardiac events are rare but have generated concern in the running community. The present results are the first to show that exercise-induced inflammation may be attenuated by statin therapy in trained, healthy runners. These results are promising for runners whose family history and/or disease pathology may place them at greater risk of a cardiac event following exercise. On the other hand, statins also increase the postexercise increase in creatine kinase suggesting that statin may enhance the muscle damage of exercise [9]. Therefore, clinicians and runners may have to weigh the risks and benefits of using statins prior to an endurance event.

## Figures and Tables

**Table 1:** Subject characteristics.

Variable	Statin (*n* = 37)	Control (*n* = 43)
Age (years)	56 ± 8	51 ± 7∗
Resting systolic blood pressure (mmHg)	140 ± 16	137 ± 17
Resting diastolic blood pressure (mmHg)	78 ± 15	78 ± 11
Body mass index (kg/m^2^)	23.6 ± 2.5	23.1 ± 2.9
Low-density lipoprotein cholesterol (mg/dL)	87 ± 26	104 ± 24∗
High-density lipoprotein cholesterol (mg/dL)	65 ± 14	74 ± 21∗
Baseline p-selectin	52.6 ± 20.8	54.7 ± 21.7
Training mileage (miles/week)	37 ± 19	40 ± 13
Taper mileage (miles/week)	22 ± 16	19 ± 11
Official finishing time (hr:min)	4:15 ± 0:47	3:58 ± 0:41
Blood pressure medication use	*n* = 9	*n* = 2
Vitamin/supplement use	*n* = 12	*n* = 14

Training mileage = average miles run per week during training for the Boston Marathon; Taper mileage = miles run in the week preceding the marathon. ^*^ *P* < 0.05; statin versus control.

**Table 2:** Types of statin drugs and doses used by number of participants.

Types of statin drugs	Doses (number of participants)			
Fluvastatin	80 mg (1)			
Atorvastatin	5 mg (2)	10 mg (3)	20 mg (6)	80 mg (1)
Rosuvastatin	5 mg (1)	10 mg (3)		
Simvastatin	10 mg (2)	20 mg (8)	40 mg (6)	
Lovastatin	20 mg (2)			
Pravastatin	10 mg (2)			

Numbers of participants taking each statin drug and dose are indicated in parentheses after dose.
